# New Antihyperglycemic Drugs and Heart Failure: Synopsis of Basic and Clinical Data

**DOI:** 10.1155/2017/1253425

**Published:** 2017-08-15

**Authors:** Dirk von Lewinski, Ewald Kolesnik, Markus Wallner, Michael Resl, Harald Sourij

**Affiliations:** ^1^Department of Cardiology, Medical University of Graz, Auenbruggerplatz 15, 8036 Graz, Austria; ^2^Cardiovascular Research Center, Lewis Katz School of Medicine, Temple University, Philadelphia, PA 19140, USA; ^3^Department of Endocrinology, Medical University of Vienna, Währinger Gürtel 18-20, 1090 Vienna, Austria; ^4^Department of Internal Medicine, Hospital Barmherzige Brüder Linz, Seilerstätte 2, 4021 Linz, Austria; ^5^Department of Endocrinology and Diabetology, Medical University of Graz, Auenbruggerplatz 15, 8036 Graz, Austria

## Abstract

The assessment of the cardiovascular safety profile of any newly developed antihyperglycemic drug is mandatory before registration, as a meta-analysis raised alarm describing a significant increase in myocardial infarction with the thiazolidinedione rosiglitazone. The first results from completed cardiovascular outcome trials are already available: TECOS, SAVOR-TIMI, and EXAMINE investigated dipeptidyl peptidase 4 (DPP-4) inhibitors, ELIXA, LEADER, and SUSTAIN-6 investigated glucagon-like peptide 1 (GLP-1) receptor agonists, and EMPA-REG OUTCOME and CANVAS investigated sodium-dependent glucose transporter 2 (SGLT-2) inhibitors. LEADER, SUSTAIN-6, EMPA-REG OUTCOME, and CANVAS showed potential beneficial results, while the SAVOR-TIMI trial had an increased rate of hospitalization for heart failure. Meanwhile, the same drugs are investigated in preclinical experiments mainly using various animal models, which aim to find interactions and elucidate the underlying downstream mechanisms between the antihyperglycemic drugs and the cardiovascular system. Yet the direct link for observed effects, especially for DPP-4 and SGLT-2 inhibitors, is still unknown. Further inquiry into these mechanisms is crucial for the interpretation of the clinical trials' outcome and, vice versa, the clinical trials provide hints for an involvement of the cardiovascular system. The synopsis of preclinical and clinical data is essential for a detailed understanding of benefits and risks of new antihyperglycemic drugs.

## 1. Introduction

**Figure 1:**
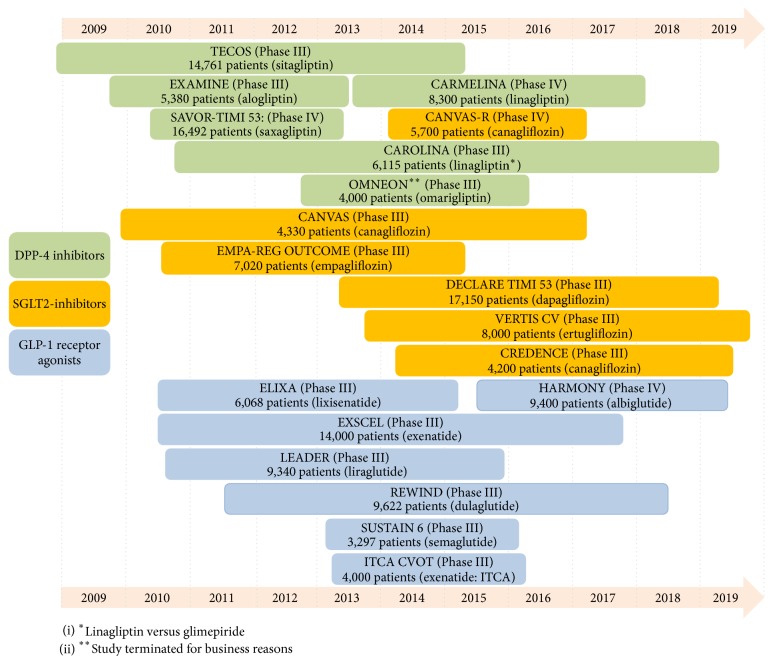
Timeline of already completed and still running cardiovascular safety trials. Green: DPP-4 inhibitors, orange: SGLT-2 inhibitors, and blue: GLP-1 receptor agonists. Name and number of planned included patients are given. All trials tested drugs versus placebo except the CAROLINA trail (linagliptin versus glimepiride).

Throughout the last decade, demonstration of glucose lowering efficacy was the primary basis for the approval of antihyperglycemic drugs. However, increasing concerns about the cardiovascular safety profile of already approved glucose lowering drugs or drugs under consideration for approval have emerged. In 2007, Nissen and Wolski published their meta-analysis describing a relative 43% increase in myocardial infarction with the use of thiazolidinedione rosiglitazone [1]. The Food and Drug Administration (FDA) and the European Medicines Agency (EMA) responded by mandating the demonstration of the cardiovascular safety profile of novel antihyperglycemic drugs, requiring a cardiovascular outcome trial [2]. This novel regulation has changed the landscape for clinical trials in the field of diabetes significantly and since 2008 more than 160,000 patients have been enrolled in cardiovascular outcome trials (Figure 1) [3]. Augmenting data on potential cardiovascular side effects of antidiabetic drugs is very valuable since millions of people are treated over many years. In most of these patients, multiple cardiovascular risk factors are commonly present, so lowering the risk for macrovascular complications is one of the major tasks in current multifactorial diabetes management. Over the last years besides the classical primary ischemic endpoints, heart failure has emerged as an increasingly important endpoint in diabetes outcome trials. Diabetes is a major risk factor for the development of heart failure [4], with approximately 22% of subjects with type 2 diabetes at an age above 65 years having a heart failure diagnosis [5].

Since 2013, eight of the FDA and EMA mandated trials have reported their results. There is no doubt that major cardiovascular events (MACE), death, and heart failure are indeed robust clinical endpoints; however, some of the results such as the potential heart failure signal for the dipeptidyl peptidase 4 (DPP-4) inhibitor saxagliptin in SAVOR-TIMI 53 or the pronounced cardiovascular benefit of the sodium-dependent glucose transporter 2 (SGLT-2) inhibitors empagliflozin and canagliflozin were rather surprising. Interestingly, there is little mechanistic insight to derive from these outcome trial data explaining cardiovascular harm or benefit. To sufficiently power these outcome trials while keeping the number of subjects and follow-up duration within acceptable limits, patients with diabetes and high cardiovascular risk or previously diagnosed atherosclerotic disease are randomized in these trials. However, the majority of patients with diabetes in routine care do not have a cardiovascular risk as high as represented by these trials [6].

This must be kept in mind, especially when findings from these outcome trials are extrapolated to patients with low cardiovascular risk. Performing outcome trials in the primary prevention setting would be important to inform future diabetes treatment, although this is a challenging task: given a MACE rate of approximately one-third as compared to subjects in the secondary prevention setting, trials in low cardiovascular risk patients would need to last longer, include more subjects, or combine both approaches, leading to a significant increase in the costs for such trials. Therefore, the synopsis of outcome data and results of basic research on cell and tissue level in models with elevated or not-elevated cardiovascular risk are of relevance and discussed in this review.

## 2. Diabetic Heart

Heart failure in diabetes represents a multifactorial problem resulting from a variety of cardiotoxic factors, such as coronary artery disease, hypertension, and direct harmful effects of glucose on the myocardium [7]. Besides well characterized macrovascular effects leading to coronary heart disease and corresponding clinical events, there is increasing data suggesting that there are direct associations between diabetes and heart failure. A 2-fold higher risk of heart failure in male diabetics and a 5-fold increase in risk in female patients with diabetes have already been demonstrated in the Framingham study [8] and this association is of particular importance in younger patients [5]. The underlying mechanisms include but are not limited to increased interstitial and perivascular fibrosis. This histological pattern was considered the basis for the term “diabetic cardiomyopathy” in the early 1970s [9]. This type of fibrosis is independent of coronary artery disease or hypertension [10]. Nonetheless, diabetic cardiomyopathy remains only moderately understood. Advanced glycation end products (AGE) [11] and increased content crosslinking of collagen seem to play a significant role [12–14]. Besides histological findings, calcium homeostasis is probably affected directly as indicated by lower activity levels of the sarco/endoplasmic reticulum Ca^2+^-ATPase 2a (SERCA2a) in diabetic hearts [15]. Moreover, SERCA2a is a major regulator of glucose transport in the healthy and diabetic heart via calcium mediated glucose transporter (GLUT) type 4 translocation [16].

There is robust evidence that metabolic abnormalities underlie the impaired myocardial function in heart failure. Metabolic parameters such as the adenosine triphosphate to phosphocreatine ratio (ATP/PCr) have been shown to predict outcome even better than left ventricular ejection fraction (LV-EF) or the clinical NYHA class [17]. In addition, changes in myocardial metabolism show direct and acute effects on mechanical performance and this effect seems to be of particular importance in human myocardium. Insulin administration itself exerts positive inotropic effects in human ventricular myocardium via Ca^2+^-dependent and Ca^2+^-independent mechanisms. Both mechanisms raise the load of the sarcoplasmic reticulum (SR) resulting in an increase of systolic Ca^2+^-transients as well as an increase in myofilament sensitivity [18]. The metabolic changes upon insulin administration could be traced back to altered GLUT-4 translocation and SGLT-1 activation [19, 20]. Additionally, insulin administration does not only result in acute functional effects, but also triggers various approaches modifying the energy substrate metabolism via an increased rate of pyruvate supply, as shown in vitro as well as in vivo [21, 22].

Heart failure and diabetes interact bidirectionally. Besides an HbA1c dependent increased risk of developing heart failure in patients with diabetes mellitus, the prevalence of diabetes in heart failure patients is known to increase markedly over time (3.8% per year) [23, 24]. Experimental data provides insight into substance-specific effects of glucose lowering therapy in heart failure. So far, with respect to the single classes of antidiabetic drugs and the related individual substances, the amount and quality of available experimental data are heterogeneous.

## 3. DPP-4 Inhibitors

While sitagliptin, alogliptin, and saxagliptin were shown to be safe for the cardiovascular system in terms of the MACE, cardiovascular death, and heart failure endpoints, the SAVOR-TIMI 53 trial showed a rather surprising signal for an increased risk for hospitalization of heart failure in the saxagliptin group, especially in the subgroups of impaired renal function and preexisting heart failure [25]. A similar trend could be observed for alogliptin in the EXAMINE trial (EXAMINE), albeit not statistically significant. In contrast, TECOS did not show an increased rate for heart failure hospitalizations after sitagliptin administration, suggesting a potential difference between members of the DPP-4 inhibitor class. The cardiovascular outcome trials CARMELINA and CAROLINA (both for linagliptin) are still running and results are expected in 2018 and 2019, respectively. Recent meta-analyses including the finished major and many smaller cardiovascular safety studies for DPP-4 inhibitors have different conclusions, ranging from no increased risk for the hospitalization of heart failure after DPP4 inhibitor use [26] to an increased risk [27].

However, studies that examine the potential pleiotropic and nonglycemic effects of DPP-4 inhibitors on various cells and tissues may help to understand and interpret the difference in the observed cardiovascular side effects in some of the clinical trials. Recently, many reviews have tried to clarify the effects caused by DPP-4 inhibitors. They interact strongly with the heart, vascular system, kidney, liver, neuroendocrine system, immune system, and hematopoietic system affecting hormones or second messengers like brain natriuretic peptide (BNP), substance P, activation of chemokine and cytokine pathways, intracellular calcium concentrations, and the release of nitric oxide (NO) shown in different animal models in vivo and ex vivo [28–32]. Interactions of DPP-4 inhibitors with the cardiovascular system and cardiomyocytes were successfully revealed, yet a direct link between DPP-4 inhibitors and its effects on cardiac contractility and/or electrophysiological function is still unknown, and the corresponding downstream mechanisms have yet to be determined. Therefore, studies that explored effects of DPP-4 inhibitors on cardiovascular system are of particular interest.

For saxagliptin, overwhelming potential beneficial effects are reported in literature: it reduces the damage of blood vessels via the amelioration of the availability of NO and the reduction of cyclooxygenase-1-action derived vasoconstriction caused by induced type-2 diabetes mellitus in mice [33] and, similarly, leads to a restoration of damaged mitochondrial vascular function in diabetic rats [34]. Additionally, a reduction of blood pressure by increasing the bioavailability of NO in spontaneous hypertensive rats [35] and an improvement of cardiac function after myocardial infarction independent of glucose lowering [36] could be demonstrated in diabetic rats. One study clarified that saxagliptin alters the cGMP-PKG-PDE5 axis in a swine model that mimicked heart failure with preserved ejection fraction (HFpEF) by aortic banding thus preventing left ventricular damage and improving left ventricular systolic and diastolic function [37]. Another study that explored the effects of saxagliptin on human multicellular myocardium and guinea pig ventricular cardiomyocytes revealed a negative inotropic potential, the prolongation of the action potential duration, and the occurrence of arrhythmias although the exact mechanism has not yet been determined [38].

Similar effects are reported for sitagliptin in diabetic rats. Sitagliptin improved endothelial function [39] and attenuated cardiac remodeling without affecting systolic function after myocardial infarction [40] while, in normoglycemic rats with induced myocardial infarction, sitagliptin prevented fatal arrhythmias by attenuating GIP-dependent resistin signaling [41] and in a PKA-dependent pathway [42]. Moreover, sitagliptin attenuated changes in the electrophysiological function in hypertensive rats [43] and counteracted induced HFpEF by improving the diastolic function, decreasing the generation of reactive oxygen species, and reducing proinflammatory biomarkers in the myocardium thus lowering mortality [44, 45]. Similarly, one study proved the reduction of parameters of diastolic dysfunction and myocardial stiffness via the cGMP-PKG pathway after sitagliptin administration in obese diabetic mice [46].

Alogliptin could restore cardiac remodeling and prevent apoptosis via a cAMP-Epac1 dependent and protein PKA-independent mechanism in a model of ventricular pressure overload [47] and inhibited inflammation in arteries that sustained damage by high LDL concentrations [48] in mice. The reported potential beneficial effects might also be present in humans; one trial with a small number of participants showed increased coronary flow reserve and improved left ventricular ejection fraction in patients with type-2 diabetes and coronary artery disease within three months of alogliptin use [49].

For vildagliptin, conflicting results are reported: one study failed to show potential protective effects on cardiac function after myocardial infarction which thereby followed cardiac remodeling despite increased levels of active glucagon-like peptide 1 (GLP-1) in rats [50]. In contrast, other studies suggested that vildagliptin might reduce infarct size and preserve left ventricular ejection fraction by reducing reactive oxygen species in a rat model of ischemia/reperfusion [51] and preventing hypertrophy of the left ventricle after continuous infusion of isoproterenol in rats by the inhibition of inflammatory markers [52]. Additionally, vildagliptin exerts effects via NO and the endothelial NO-synthase (eNOS) leading to an improved vascularization in a mouse model with surgical induced ischemia [53]. Focusing on the cardiovascular system, vildagliptin seems to exert similar effects as sitagliptin [54]. However, no large cardiovascular outcome trial for vildagliptin is being performed.

Finally, linagliptin improves diastolic function in a model of HFpEF in obese rats via an elevated expression of eNOS and improved SERCA2a activity [55]. The effect on eNOS availability could be demonstrated in nonobese mice as well [56]. Linagliptin also reduced angiotensin and glucose induced collagen formation in cardiac fibroblasts of mice by an anti-inflammatory mechanism (via NFkB) [57].

## 4. GLP-1 Receptor Agonists

The first cardiovascular outcome trial on glucagon-like peptide-1 receptor agonists was the ELIXA trial, which was designed to assess the effects of lixisenatide on the cardiovascular outcome in patients with type-2 diabetes mellitus who had an acute coronary event within 180 days of screening. For the primary composite endpoint (cardiovascular death, myocardial infarction, and stroke), as well as for hospitalization for heart failure, no significant difference was observed between the treatment and placebo group [58]. The LEADER trial assessed the cardiovascular safety of liraglutide in patients with type-2 diabetes mellitus and a HbA1c ≥ 7%. Of the total enrolled subjects, 81.3% had preexisting cardiovascular diseases. Liraglutide significantly reduced the rate of the first occurrence of the primary endpoint (cardiovascular death, nonfatal myocardial infarction, or nonfatal stroke) and all-cause mortality. The rates of nonfatal stroke, myocardial infarction, and hospitalization for heart failure were nonsignificantly lower in the liraglutide group compared to the placebo group [59]. In the SUSTAIN-6 trial (semaglutide) patients with type-2 diabetes mellitus and established cardiovascular diseases, chronic heart failure, or chronic kidney disease, or ≥60 years with at least one cardiovascular risk factor, were enrolled. Semaglutide significantly reduced the risk for the primary endpoint (first occurrence of cardiovascular death, nonfatal myocardial infarction, or nonfatal stroke). The protective effect of semaglutide on composite endpoints seems to be mainly driven by the reduction of nonfatal stroke [60]. The results of LEADER and SUSTAIN-6 continue to hold promise that GLP-1 receptor agonists might improve CV morbidity in patients with type-2 diabetes mellitus. However, we do not yet fully understand the reasons for the diverging results in the currently published trials. Differences in the duration of action (short acting substances such as lixisenatide versus longer acting drugs like liraglutide or semaglutide) or differences within the amino acid sequences of the peptides are currently being discussed. Further insight will be gained from the imminent presentation of the EXSCEL trail [61].

GLP-1 is an incretin peptide hormone primarily synthesized by intestinal L cells [62]. It is released into the circulation in response to food intake, leading to glucose-dependent insulin release and glucagon suppression. GLP-1(7–36)NH_2_, with a half-life of 2 minutes, is the primary active isoform that is rapidly degraded by DPP-4 to GLP-1(9–36)NH_2_ [63], a GLP-1 receptor antagonist [64]. Besides increased insulin secretion, GLP-1 receptor activation leads to an inhibition of gastric and small bowel motility, reduces appetite, and subsequently leads to weight loss [65]. In addition, human data suggests that this drug class improves cardiac function in patients with congestive heart failure, ameliorates endothelial dysfunction, and reduces the infarct size after ST-segment-elevation myocardial infarction [66–69]

The GLP-1 receptor is a seven transmembrane, G protein-coupled receptor (GPCR), and is positively coupled to adenylate cyclase through G*α*_s_-containing G proteins, which catalyze the conversion of ATP to cAMP. Increased cytosolic cAMP leads to activation of second messenger pathways including PKA, Epac2, and ERK-1/2 [70]. Beneficial effects of GLP-1 receptor agonists have been attributed to direct action on myocardium, with the majority of these effects reported in ventricular cardiomyocytes. However, there are conflicting reports regarding GLP-1 receptor expression in cardiac tissue. Recent studies in mice and rats revealed that the GLP-1 receptor is exclusively localized in atrial cardiomyocytes [71–73]. Wallner et al. reported GLP-1 receptor expression in human right and left ventricular myocardium, although the expression levels were significantly lower compared to right atrial tissue [74]. This discrepancy between human and rodent tissue could be explained by species-related differences, such as those that exist for the SGLT, which is expressed in human myocardium but is undetectable in the myocardium of most species [20].

A recent study in normo- and hypertensive mice suggested that GLP-1 receptor activation in atrial cardiomyocytes increased cAMP levels, promoted Epac2 translocation to the membrane, and increased ANP secretion [71]. Epac2 functions in a PKA-independent manner and, therefore, represents a novel mechanism for governing signaling specificity within the cAMP cascade [75]. A recent study reported significant Epac2 translocation from the cytosol to the cell membrane after GLP-1 receptor activation in human atrial myocardium [74]. Epac2 activation increases phosphorylation of cardiac troponin I (cTnI) in a PKC-dependent manner resulting in increased myofilament Ca^2+^ sensitivity and contractility [76]. GLP-1 receptor agonists significantly increased developed force in human atrial trabeculae, whereas Exendin(9–39)NH_2_, a GLP-1 receptor antagonist, and H-89, a PKA inhibitor, blunted the inotropic effect of exenatide. In addition, exenatide (a synthetic GLP-1 receptor agonist that is resistant to the degradation by DPP4) increased PKA-dependent phosphorylation of phospholamban (PLB) and GLUT-1 translocation, but not GLUT-4 translocation [74]. *β*-Arrestin signaling downstream of GLP-1 receptor activation is another potential mechanism to increase cardiac contractility. *β*-arrestin, which is well-known for contributing to the termination of GPCR signaling [77], might regulate cardiac function and increase cardiac contractility via *β*-arrestin-mediated processes [78–80]. Novel “biased ligands” that selectively recruit *β*-arrestin independent of G protein-mediated signaling have been described for the angiotensin II Type 1A receptor (AT1AR) [78] and the *β*1-adrenergic receptor (*β*1AR) [80]. However, Wallner et al. showed that ß-arrestin signaling downstream of GLP-1 receptor activation does not contribute to the positive inotropic effect in human atrial myocardium [74].

## 5. SGLT-2 Inhibitors

In 2015, the EMPA-REG-OUTCOME trial demonstrated a significant reduction in MACE and all-cause mortality in subjects treated with the SGLT-2 inhibitor empagliflozin [81]. Moreover, this landmark trial showed a 35% relative reduction in the rate of heart failure hospitalization in the empagliflozin group, an effect occurring very quickly after initiating treatment. These findings on MACE and heart failure hospitalization were confirmed in the recently published data from the CANVAS program with canagliflozin [82]. However, cardiovascular and all-cause mortality were not significantly reduced by canagliflozin, in contrast to empagliflozin. Currently, several hypotheses are being discussed for the findings in the SGLT-2 inhibitor trials. These include hemodynamic changes and increased hematocrit that are caused by a diuretic effect or changes in the cardiac fuel metabolism by an improved uptake of *β*-hydroxybutyrate under conditions of persistent hyperketonemia, all induced by SGLT-2 inhibitors. Particularly ischemic and therefore endangered myocardium may benefit from these effects [83, 84].

For SGLT-2 inhibitors, the most recent class of antidiabetic drugs established for clinical use, there is little data on cardiovascular side effects in animal models or in vitro settings available. This may be a consequence of the fact that the SGLT-2 receptor is not expressed in myocardial tissue [20, 81, 85]. Mechanistically, cardiovascular side effects of SGLT-2 inhibitors could occur either via unselective binding of compounds to SGLT-1, which is not the case for most of the members of this drug class, or via receptor independent effects. Interestingly, the pattern of intracellular mechanisms seems to be different for various class members.

Activation of AMPK, for example, has only been shown for canagliflozin but not for dapagliflozin or empagliflozin [86]. However, a pathway most likely influenced by all SGLT-2 inhibitors in cardiomyocytes is the Na^+^/H^+^ exchanger 1 (NHE1) mediated decrease in intracellular Na^+^ and Ca^2+^, although this has only been reported for empagliflozin so far. Decreased intracellular Ca^2+^ is likely to result in a negative inotropic effect; however, this is not necessarily the case if both systolic and diastolic Ca^2+^ decrease and the Ca^2+^ transient remains stable. Moreover, Baartscheer et al. did show that mitochondrial Ca^2+^ ([Ca^2+^]_m_) did significantly increase upon empagliflozin administration. [Ca^2+^]_m_ signaling is critical for energy production as well as the activation of cell death pathways which are implicated in the development of heart failure [87]. These three changes in intracellular ion homeostasis counteract the alterations typically seen in heart failure models (e.g., elevated levels of intracellular Na^+^ and Ca^2+^ and reduced levels of mitochondrial Ca^2+^ in heart failure) and might thus explain at least in part beneficial effects as seen in the EMPA-REG-OUTCOME trial [81].

Elevated diastolic Ca^2+^ also results in impaired relaxation and therefore diastolic dysfunction. Interestingly, empagliflozin significantly improved diastolic function in a rodent model of diabetes and reduced the expression of profibrotic and prohypertrophic proteins [88]. These effects could not be explained by reduced blood pressure levels as reported in several other models after SGLT-2 treatment, indicating towards a direct myocardial effect.

This idea is also supported by the finding that dapagliflozin but not pioglitazone significantly improves cardiac function in a mouse model despite comparable glucose lowering effects. Ejection fraction and isovolumetric relaxation time were not altered in pioglitazone, but E/A ratio and ventricular hypertrophy were both slightly improved.

## 6. Metformin

In the United Kingdom Prospective Diabetes Study (UKPDS), 342 patients with an ideal body weight greater than 120% were randomly assigned to an intensive treatment with metformin or conventional treatment. A 39% relative risk reduction in fatal and nonfatal myocardial infarction (*p* = 0.010) and a 36% relative risk reduction in all-cause mortality (*p* = 0.011) were recorded in this study arm [89]. This finding in a limited number of patients is supported by data from a meta-analysis performed with randomized clinical trials data [90] suggesting a cardiovascular benefit associated with metformin. A larger trial investigating the effect of metformin in nondiabetic hyperglycemia is currently ongoing (ISRCTN 34875079).

## 7. Conclusion

**Figure 2:**
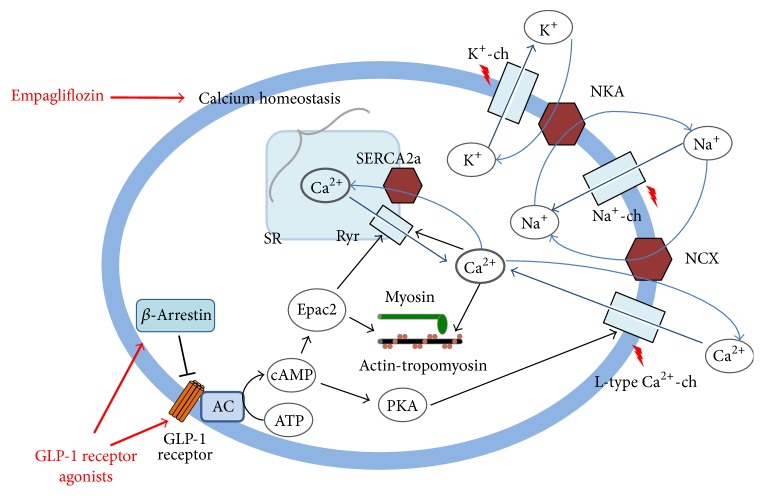
Interactions of antidiabetic drugs with cardiomyocytes: the well-established downstream mechanism of GLP-1 receptor agonists alters intracellular Ca^2+^ transients via a PKA-dependent activation of L-type Ca^2+^ channels and Epac2-dependent activation of the ryanodine receptor. For empagliflozin, potential downstream mechanisms are still unknown, yet there is strong evidence that the Ca^2+^ homeostasis is influenced. Possible downstream mechanisms of DPP-4 inhibitors are also still unknown. The wide interactions with cardiomyocytes via miscellaneous second messengers are not shown. (Na^+^-ch: voltage gated sodium channel, K^+^-ch: voltage gated potassium channel, Ryr; ryanodine receptor, NCX: sodium-calcium exchange pump, and NKA: sodium-potassium exchange pump).

With new and emerging primarily antihyperglycemic drugs, the intersection of antidiabetic treatment and cardiovascular therapy is progressing. Besides modulating diabetes as a cardiovascular risk factor several new antidiabetic drugs imply direct cardiovascular effects and in some cases these effects seem to directly affect myocardial tissue (Figure 2). Cardiovascular outcome trials requested by the FDA and EMA were designed to test for global and rather indirect cardiovascular effects and the mechanistic basis for the beneficial findings in some of these trials remain to be elucidated. Although these trials are called placebo-controlled trials, subjects in the control arm receive usual diabetes care excluding compound/drug class used in the active arm and all these trials aim for glycemic equipoise in both groups in order to exclude cardiovascular effects which could be due to differences in glycemic control [91].

Global assessment of cardiovascular outcome in usually short- to mid-term trials in particular high risk populations, however, might miss distinct intracellular effects of new antidiabetic drugs that could mediate more specific, positive or negative, effects on excitation-contraction coupling, contractility, metabolism, or energetics resulting in altered structural or functional properties.

An in-depth examination of cardiovascular outcome data in conjunction with basic science data is critical for a detailed understanding of benefits and risks of new antihyperglycemic drugs.

## Abbreviations

DPP-4: Dipeptidyl peptidase 4
EMA: European Medicines Agency
eNOS: Endothelial NO-synthase
FDA: Food and Drug Administration
GLP-1: Glucagon-like peptide 1
GLUT: Glucose transporter
HFpEF: Heart failure with preserved ejection fraction
MACE: Major adverse cardiac event
NO: Nitric oxide
SERCA2a: Sarco-/endoplasmic reticulum Ca^2+^-ATPase
SGLT: Sodium-dependent glucose transporter.
